# Beyond Microbial Variability: Disclosing the Functional Redundancy of the Core Gut Microbiota of Farmed Gilthead Sea Bream from a Bayesian Network Perspective

**DOI:** 10.3390/microorganisms13010198

**Published:** 2025-01-17

**Authors:** Federico Moroni, Fernando Naya-Català, Ahmed Ibrahem Hafez, Ricardo Domingo-Bretón, Beatriz Soriano, Carlos Llorens, Jaume Pérez-Sánchez

**Affiliations:** 1 Institute of Aquaculture Torre de la Sal (IATS-CSIC), 12595 Ribera de Cabanes, Spain; fernando.naya@iats.csic.es (F.N.-C.); ricardo.domingo@csic.es (R.D.-B.); 2 Biotechvana, Parc Científic Universitat de València, 46980 Paterna, Spain; ahmed.hafez@biotechvana.com (A.I.H.); beatriz.soriano@biotechvana.com (B.S.); carlos.llorens@biotechvana.com (C.L.)

**Keywords:** fish gut microbiome, core microbiota, processed animal proteins, functional redundancy, taxonomical variability, microbiota biomarkers, Bayesian network, bacterial functions

## Abstract

The significant microbiota variability represents a key feature that makes the full comprehension of the functional interaction between microbiota and the host an ongoing challenge. To overcome this limitation, in this study, fish intestinal microbiota was analyzed through a meta-analysis, identifying the core microbiota and constructing stochastic Bayesian network (BN) models with SAMBA. We combined three experiments performed with gilthead sea bream juveniles of the same hatchery batch, reared at the same season/location, and fed with diets enriched on processed animal proteins (PAP) and other alternative ingredients (NOPAP-PP, NOPAP-SCP). Microbiota data analysis disclosed a high individual taxonomic variability, a high functional homogeneity within trials and highlighted the importance of the core microbiota, clustering PAP and NOPAP fish microbiota composition. For both NOPAP and PAP BNs, >99% of the microbiota population were modelled, with a significant proportion of bacteria (55–69%) directly connected with the diet variable. Functional enrichment identified 11 relevant pathways expressed by different taxa across the different BNs, confirming the high metabolic plasticity and taxonomic heterogeneity. Altogether, these results reinforce the comprehension of the functional bacteria–host interactions and in the near future, allow the use of microbiota as a species-specific growth and welfare benchmark of livestock animals, and farmed fish in particular.

## 1. Introduction

The microbiome represents a complex environment inhabited by a large number of microorganisms such as bacteria, archaea, virus and eukaryotes that form a dynamic equilibrium occupying different ecological niches or organism compartments [1,2]. The last decade has witnessed an explosion in microbiome research, but our understanding of the structure and function of the microorganisms inhabiting the host and its surroundings is still limited due to the personalized nature and complexity of the microbiota and host interactions [3,4,5]. Certainly, according to the holobiont theory, the microbiota and host associations represent a synergistic system that co-evolved over time towards homeostatic loads driven by mutualism and cooperation [6]. At the same time, the microbiota offers a great functional redundancy that makes the host and microbiota associations from humans, and rodents to other animal models challenging and sometimes controversial, shaping variations in a broad range of metabolic, immunological, behavioral and reproductive host traits [7,8,9,10,11]. This also applies to farmed fish, in which the manipulation of the gut microbiome has the potential to generate substantial improvements in aquaculture production, that has reached 88 million tons in 2020 with a sale value of USD 265 billion [12,13,14,15].

The great variability in bacterial communities inhabiting the gut intestine of living animals, and farmed fish in particular, remains an ongoing challenge that is difficult to solve and exploit without considering the combined effects of host genetics and diet. Indeed, both in gilthead sea bream and European sea bass farmed fish, there is now evidence that the gut microbiota variability is decreased by selective breeding and improved by feed formulations for enhanced growth [16,17]. However, in genetically selected fish, both the inferred metagenome and wide meta-transcriptomics approaches have revealed a functionally more plastic microbial community, which does not require significant alterations in composition to cope with changes in diet [18,19]. Such observations harbor clear host genome and metagenome interactions that are also concurrent with a more responsive host transcriptome in fast-growing families, which would become decisive for the effective function and regulation of the holobiont system [20]. However, univocal changes in fish gut microbiota are mostly limited to the phylum level, characterized by a high over-representation of Pseudomonadota (formerly Proteobacteria), Bacteroidota (formerly Bacteroidetes) and Bacillota (formerly Firmicutes) [21,22,23], while it remains challenging to solve its functional redundancy in both fish and humans [24]. A strategy through which this topic can be approached is identified via the core microbiota, defined as the most stable and/or abundant microbial population that would also represent the most ecologically and functionally important microbial associates [25,26,27].

According to the above findings, the definition of core microbiomes may assist in addressing the efficacy of a wide range of dietary and environmental interventions, as well as a proxy for the early indicators of dysbiosis or inflammation of the gastro-intestinal tract (GIT), often characterized by a multi-factorial etiology [28]. Thus far, several attempts have been made using this definition, but the significant individual variability, together with the technical limitations due to a limited number of samples, sequencing depth, and poorly predictable environmental factors, make it difficult to establish a robust and commonly accepted core microbiota at a lower taxonomic level [29,30]. In any case, the identification of the most abundant fraction of the microbiota populations is not sufficient to fully understand its functional profile and contribution within the host’s metabolism. The introduction of statistical learning-based models is in fact necessary to better identify the complex features within microbiota studies. Thus, the use of Bayesian network (BN) platforms, such as the recently published SAMBA [31], is becoming a useful tool for investigating the close directional relationships within the social network organization that characterize bacterial populations, but also how these microbial associations can be modulated by one or more experimental/environmental variables [32]. However, in contrast to the networks built for macro-organisms that are constructed using functional and ecological macroscopic features that are easy to be measured, the microbial networks are commonly structured only using sequencing data [33]. Such a technical limitation represents a potential drawback that numerous Bayesian network (BN) approaches are partially or not properly considered [34,35,36]. Therefore, there is a huge need to develop new instruments to go deeper into the functionality of microbiota, and to transform bacterial taxa into functional units rather than simple changes in the profile composition.

In this context, to address the challenges in studying the microbiome, the aim of the present work was to establish a robust core microbiota that would serve to re-think the taxonomic variability and functional redundancy of the resident gut microbiota of gilthead sea bream using a new perspective. To achieve these goals, information from previously published gilthead sea bream feeding trials [37,38,39] was used herein as a unified starting matrix. Such an approach allowed us to evaluate in juvenile fish the effect of different dietary protein sources on the gut-associated bacterial community of fish with the same genetic background, kept in the same experimental facility for the entire duration of the trials. Through this multi-trial approach, we aimed to modify the classical idea of microbiota biomarkers into a new paradigm that combines the following multiple factors: (i) taxonomic classification, (ii) role in the bacterial network and (iii) functional contribution to the binomial host–microbiota organization. Such a methodology does not only represent an exclusive advance in aquaculture, but rather it can be widely applied to other microbiome studies, where an ecological–functional analysis of the environment or holobiont system is required.

## 2. Materials and Methods

### 2.1. Experimental Microbial Datasets

The three microbiota datasets used in this study originate from feeding trials conducted in parallel (spring–summer 2020) at the indoor marine research infrastructure of the Institute of Aquaculture of Torre de la Sal, using a marine flow through system under natural light and temperature conditions (40°5′N; 0°10′ E). All fish had the same origin (AVRAMAR, Burriana, Spain) and were fed close to the visual satiety with the following different diet formulations: (i) a plant-based diet supplemented with an of egg white hydrolysate preparation [37]; (ii) a free-fish meal diet with a high inclusion level of insect meal and fermented biomass of single cell proteins (SCP) [39] and (iii) a fish meal-replacer of processed animal proteins (PAPs) and SCPs, for a partial (50%) or total (100%) fish meal replacement [38]. According to these feed formulations, the resulting fish groups were re-named as NOPAP-PP, NOPAP-SCP and PAP, respectively (Table S1). In all cases, the amplicon sequencing targeting the hypervariable 16S rRNA gene with V3–V4 primers was used for the analysis of the microbiota composition using the Illumina MiSeq system (Illumina Inc., San Diego, CA, USA) at the Genomics Unit from the Madrid Science Park Foundation (FPCM, Campus de Cantoblanco, Madrid, Spain). Up to 844 Operational Taxonomic Units (OTUs) were assigned using the Ribosomal Database Project (RDP). Bacterial taxonomy was then updated according to SILVA v138.1 database, one of the most referenced and updated 16S rRNA databases. Sample depth was normalized by total sum scaling and then made proportional to the total sequencing depth [40].

### 2.2. The Inferred Metagenome

The analysis of the functional profile of the three gut microbiota datasets was performed by uploading the taxa raw counts to the SAMBA platform, and the fasta file with all the sequences associated to a given bacterial taxon. As already described [31], the inferred metagenome analysis was performed using PICRUSt2 protocol, assigning metagenomic functions using the Kyoto Encyclopedia of Genes and Genomes database (KEGG) [41]. To evaluate the possible differences in significantly impacted pathways due to the nutritional intervention, pathway analysis was performed independently within each experimental feeding trial.

### 2.3. The Bayesian Network Construction

All the data from the three microbial datasets were used to perform a functional meta-analysis of the gut microbiota using a BN approach. For this purpose, the construction of several BNs was carried out using the SAMBA tool. In brief, the BNs were built under the following parameters: bacterial taxa were normalized using Equation (1); taxa with a zero total counts of normalized data were removed; to fit the model, the Zero-inflated Negative Binomial (ZINB) distribution of the normalized microbial abundances was used and the strength of each connection (edge) in the model was calculated using Bayesian information criterion (BIC) and mutual information (MI) criterion, fixing the threshold at MI < 0.05 and BIC < 0. To further deepen the analysis, once the BN was obtained, the identification of clusters of nodes densely connected was performed using the Leiden community detection method [42]. Then, to infer their metabolic and functional profile, for each cluster, an enrichment was carried out using the clusterProfiler 4.0 R package [43] based on the KEGG database for the pathway functional annotation protocol.

(1) $$NC_{ij}=\frac{X_{ij}\times \sum_{n=1}^{N}X_{in}}{\left(\sum_{t=1}^{T}X_{tj}\right)}$$

where NC_ij_ is the normalized count for a taxon i in a concrete sample j; X_ij_ is the raw counts of a concrete taxon i in a specific sample j; N is the number of samples in the dataset and T is the number of taxa in the dataset.

### 2.4. Statistical Analysis

The identification of statistical differences between the experimental groups in the taxonomic bacterial profile was determined by the Kruskal–Wallis test, with a significant threshold of *p* < 0.05, while the differences regarding the inferred metagenome analysis were performed with the R Bioconductor package DESeq2 (version 1.42.1) using default parameters. The metagenomic pathways were considered differentially represented using an FDR corrected significance threshold (*p*-adjusted) of 0.05. The comparison between the bacterial profiles of the three different bacterial datasets was analyzed by partial least-squares discriminant analysis (PLS-DA) using EZinfo v3.0 (Umetrics, Umeå, Sweden). The outlier’s identification was performed using Hotelling’s T2 statistic, setting a 95% confidence limit for T2. The quality of the PLS-DA model obtained was evaluated by the parameters R2Y (cum) and Q2 (cum). A validation test of the PLS-DA model was performed using the Bioconductor R package ropls, consisting of 500 random permutations. The OTUs that most contributed to group separation were determined by the minimum variable importance in the projection (VIP) values, using a VIP threshold ≥ 1.2. The analysis of the significance of the functional enrichment of the BN clusters was calculated with clusterProfiler package, using a hypergeometric test. This statistical test evaluated whether the number of selected OTUs in each cluster, and enriched for a given pathway, is greater than what would be expected by chance. The background data used for this comparison were taken from the list of OTUs and sequences obtained so far by the group of Nutrigenomics and Fish Growth Endocrinology of the Institute of Aquaculture Torre de la Sal (IATS-CSIC), Spain (approximately 20k sequences). Significance values were then adjusted for multiple testing using the Benjamini–Hochberg method to control the false discovery rate (FDR), ensuring that the likelihood of type I errors was minimized across the tests conducted.

## 3. Results

### 3.1. The Discordance of Taxonomical and Functional Microbiota Profiles

**Figure 1:**
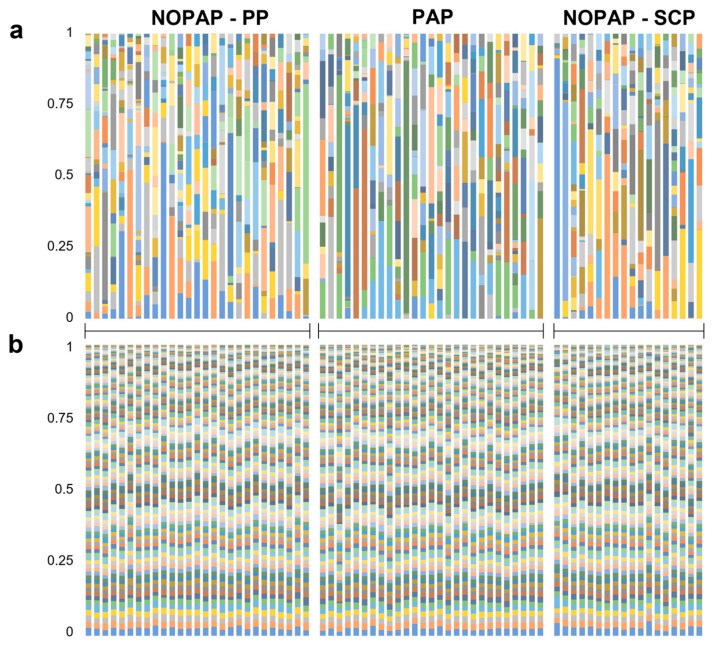
This is a stacked bar chart representing the taxonomic profile (**a**), reported as relative abundance of bacterial genera, and the inferred functional profile (**b**), reported as the level 3 KEGG metabolic pathways. Each column represents each sample considered. Samples are grouped according to the feeding trials (NOPAP-PP, PAP, NOPAP-SCP).

For assessing the differences between the taxonomical and functional profile of the intestinal microbiota composition, an inferred metagenome analysis was performed. Considering each feeding trial separately, the results showed that at a lower taxonomic level, the microbiota populations exhibited a high variability not only among the experimental groups (Table S2), but also considering an inter-sample evaluation. In fact, Figure 1a illustrates the high heterogeneity at a genus level of the taxa distribution along the different samples, and the impossibility of delineating a common pattern within or between the three trials.

Conversely, the profiles obtained by the inferred metagenome defined a metabolic pathways distribution, which clearly suggests a conserved pattern of functions (Figure 1b; KEGG pathways level 2). The same organization scheme is achieved regardless of the level defined by the KEGG database. This conserved functional distribution is confirmed by the absence of significant differences in the potential metabolic capacity between groups within the same trial for each one of the recognized pathways (see Table S3).

### 3.2. Discriminant Analysis Unveiled the Core Discriminant Microbiota

**Figure 2:**
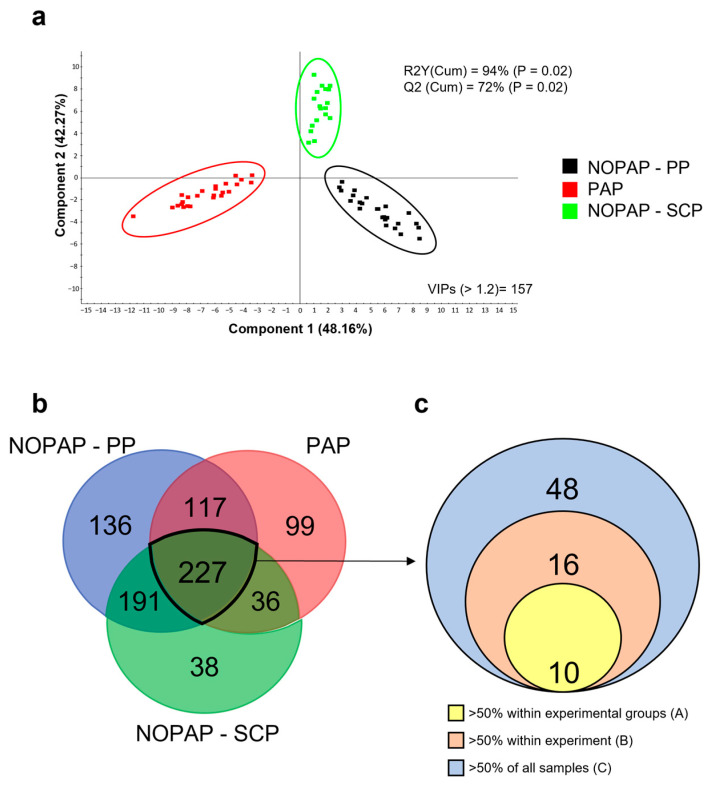
The two-dimensional PLS-DA score plot represents the distribution of the samples between the first two components in the model of the NOPAP-PP, PAP and NOPAP-SCP feeding trials. Four samples belonging to the NOPAP trial were excluded from the model because considered as outliers (**a**). Venn diagram reporting unique and shared taxa considering the total intestinal microbiota datasets of the three feeding trials (**b**). Concentric circle nested diagram representing the core microbiota within the 227 common taxa identified, divided by the rank of restrictiveness of the applied filter (**c**).

To further confirm the different microbial profile among the three feeding trials, a multivariate analysis was performed and statistically validated by a permutation test (Figure S1). The resulting PLSDA model (Figure 2a) showed a clear separation of the three experimental groups along the two components, which together account for 94% of the observed variance (R2Y (cum), *p* < 0.02) and 87% of the predicted variance (Q2 (cum), *p* < 0.02). The separation between the three groups was driven by a total of 157 bacteria having a VIP value > 1.2. Between all the datasets considered, the number of bacteria shared by all the three experiments was 227 (Figure 2b). From this group of common taxa, the core microbiota was calculated using an increasing restriction gradient filter. The results, shown in the concentric circle nested diagram (Figure 2c), highlighted 48 bacteria that were represented in more than 50% of all the analyzed samples (C), 16 of which were present in more than 50% of samples of each experiment (B) and 10 bacteria which account for more than 50% of the sample of each experimental group within each trial (A). The complete list of core microbiota, which together represent the average of 45% of the total bacterial population, is detailed (average abundance in each experiment/total abundance) in Table S4.

### 3.3. Independent Bayesian Network Meta-Analysis Revealed Reliable Gut Microbiota and Diet Associations

**Figure 3:**
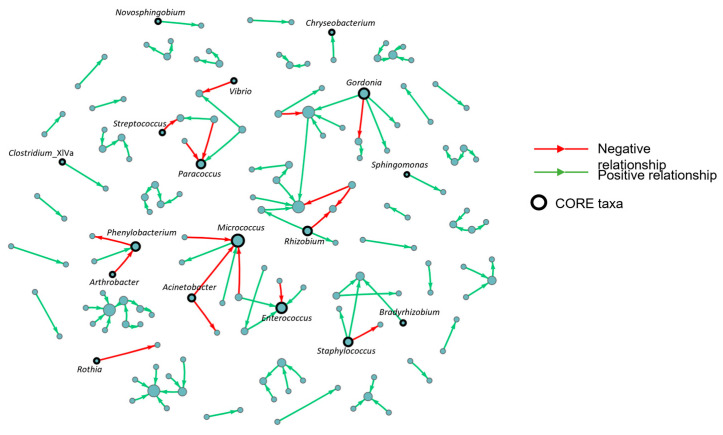
Bayesian network constructed using the three feeding trial merged datasets. The model only reports the microbial interactions, obtained by the taxa counts distribution. Green arrows represent positive interactions between nodes, while red arrows represent negative dependences. The core microbiota nodes are represented by the circles with the outline in bold in the figure.

To identify the positive and negative causal connections within the bacterial population, a meta-analysis was carried out through the construction of a stochastic BN using the merged dataset taken from the combination of the three feeding trials. The structure was designed to only focus on the microbial interactions using the taxa counts distribution, without considering the potential contribution of diet as a qualitative experimental variable. The resulting network (Figure 3) was composed of a total of 413 nodes involved in 117 edges and grouped in 41 clusters, after the application of the Leiden community detection method.

**Figure 4:**
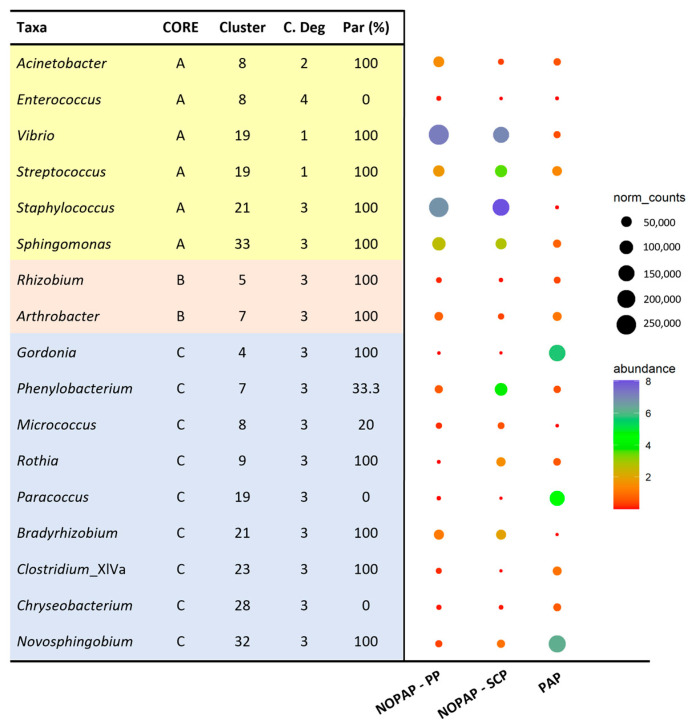
Dot plot representing the core microbiota taxa which actively participate in the edges of the BN reported in Figure 3. In the table, the core sub-groups are reported, as indicated in Figure 2c, the belonging cluster in the BN, the centrality degree (C. Degree), which represents the total number of edges where the node is involved and the percentage of parent (Par %), which indicates the percentage that the node plays in the role of parent compared to the total of its connections. Taxa are colored depending on the core sub-groups, as indicated in Figure 2c. In the dot plot, color scale represents the mean relative abundance, in percentage, of each taxon within each group. The size of the dots represents normalized counts in each group.

From the 48 bacteria identified as core microbiota, only 17 taxa were highly interconnected within the network reported in Figure 4, following the previously established categories (A; B; C). Within this list, 70% of the nodes in total act as parents (100% Par) in the edges in which they are involved. This aspect confers great importance on the core microbiota as it can influence a large portion of the remaining bacterial population. In addition to its role in the network, Figure 4 also reports the average abundance and the normalized counts of the core microbiota. As already indicated, these bacteria together represent an abundant fraction of the microbiota, and so they reflect a valid sample of the whole prokaryotic community associated with each trial. From this perspective, the tendency exhibited in the three different abundance profiles was to cluster together the NOPAP-PP and NOPAP-SCP microbiota patterns, while the PAP group emerges as a different taxa count distribution. This feature confirms the strong influence of the diet on gut microbiota, bringing together the two experimental groups that share a more similar feed composition among the three feeding trials.

### 3.4. Combined Bayesian Network Analysis Disclosed a Highly Interconnected Core Microbiota with Changes in Diet Composition

**Figure 5:**
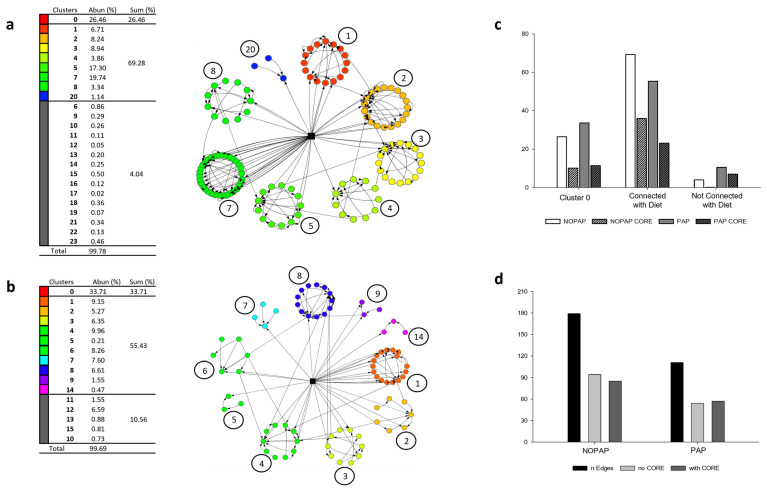
Bayesian networks representing NOPAP (**a**) and PAP (**b**) models. Circles represent bacterial taxa and squares represent the experimental variable (Diet). The tables report numbers and colors of the clusters (cluster 0 and the clusters colored in grey are not represented in the figure), the relative abundance of the taxa composing each cluster (Abun %) and the sum of the relative abundances according to the following groups: cluster 0; clusters connected to the variable, clusters not connected to the variable (Sum %). The bar plot representing the number of core microbial taxa compared to the total number of nodes belonging to the three categories already defined, for both the models, NOPAP and PAP BNs (**c**). The bar plot representing the number of edges in which the core microbiota is involved or not, compared to the total number of dependencies, for both the models (**d**).

Following the dietary-driven effects upon the core gut microbiota, the construction of two different BNs was considered. The first BN, defined as NOPAP, combined the microbiota datasets of the NOPAP-PP and NOPAP-SCP trials (Figure 5a); while the second BN, defined as PAP, only referred to the PAP-SCP trial (Figure 5b). Contrary to the model reported in Figure 3, these networks were constructed using the qualitative variable “Diet”, which allowed information to be obtained on the distribution of taxa with respect to their dependence on the experimental variable. The complete list of nodes and edges for both BNs is reported in Table S5. The two constructed BNs present the same structure, as follows: the Cluster 0, which contains the nodes isolated from the network and without connections with it; clusters of nodes which are involved in edges, but not directly/indirectly connected with the experimental variables, and only represent a range from 4 to 10% of the total number of edges (grey clusters in Figure 5) and clusters which present a direct/indirect connection with the variable “Diet”, which represent the rest of the total edges (the BN reported in Figure 5). According to all this, in the network, the modelled gut microbiota almost covers 100% of the taxa abundances (99.78 and 99.69% for NOPAP and PAP BNs, respectively). Within this percentage, the nodes connected to the experimental variable represent the majority, accounting for 69.28% in NOPAP BN and 55.43% in PAP BN; meanwhile, the bacteria not connected to the main structure only represent a minor fraction of the population. Within the two BNs, Figure 5c, d report the presence of the core microbiota in the nodes and in the edges, respectively. These results show that for both networks, the ratio between core taxa and the total number of nodes remains almost constant in Cluster 0 and in the nodes connected to the variable. Instead, in the group of the taxa not connected to the Diet, the core bacteria only represent the 3% in the NOPAP BN, while they constitute 66% of the total in the PAP BN. Regarding the edges, the proportions of core taxa were the same between the two models.

### 3.5. Functional Redundancy Analysis

The results of the functional enrichment analysis of the clusters recognized by the NOPAP and PAP BNs is reported in Table 1. Up to four and eight clusters exhibit a significant function enrichment in NOPAP and PAP BNs, respectively. These results suggest that groups of highly inter-connected bacteria can determine functional specialization, but at the same time confirms the functional redundancy of microbiota, as different clusters within the same population can express the same function.

**Figure 6:**
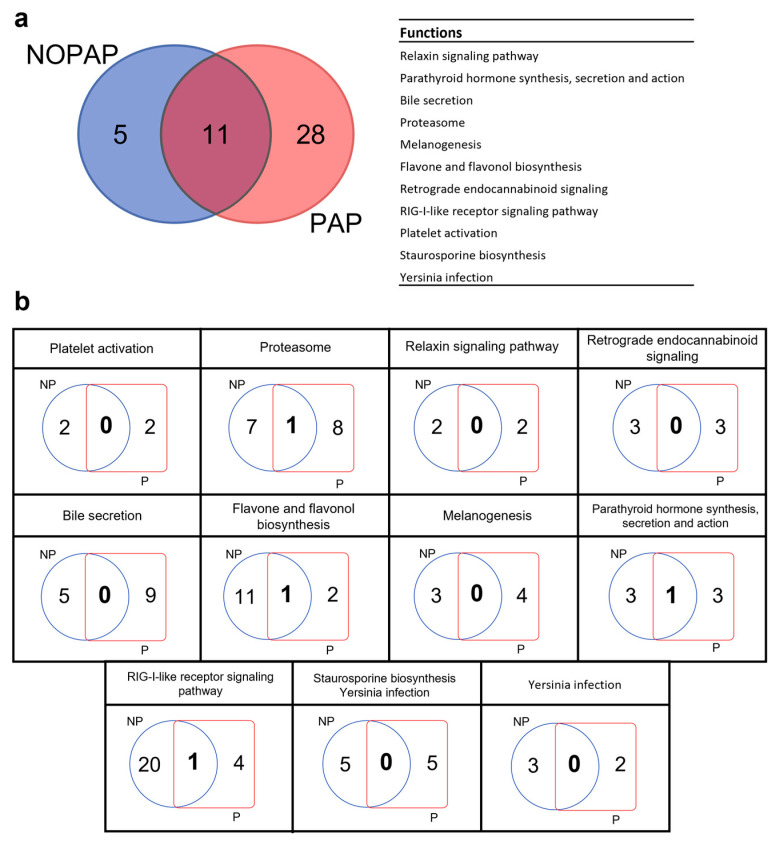
Venn diagram and table reporting the common functions expressed by both NOPAP and PAP models (**a**). Venn diagrams reporting the number of taxa responsible for each function in the two BNs of NOPAP (NP) and PAP (P) (**b**).

This feature was further corroborated by the comparisons made between the two trial networks. In fact, as reported in Figure 6a, from the total of 44 metabolic functions identified, only 11 were significantly expressed by both models, and included relaxin signaling pathways, parathyroid hormone synthesis, bile secretion, flavone and flavonol biosynthesis, endocannabinoid signaling pathway and staurosporine biosynthesis, among others. For each shared function, the number of bacteria involved in its expression was analyzed. The results underlined how there is an almost total lack of taxonomic overlap between the two profiles in the expression of the same function. In fact, as illustrated in Figure 6b, less than 4% of the bacteria that appear in both models are involved in the same metabolic pathway.

**Figure 7:**
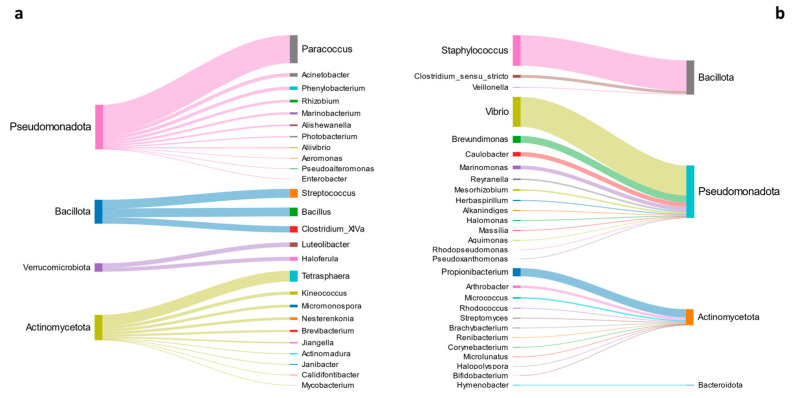
Sankey diagrams reporting the list of genera and Phyla which express the 11 inferred metabolic functions, associated to the NOPAP (**a**) and PAP (**b**) conditions.

Apart from this small fraction, the list of unique bacteria at genus level that expressed a significant enrichment in the two BN models are shown in Figure 7. Although the influence of the diet can modify the microbiota structure enhancing its functional plasticity, which is reflected in the higher variability at a low taxonomical level, at a higher classification level (Phylum), the distribution of taxa follows the normal gut microbiota profile of gilthead sea bream, characterized by the major abundance of Pseudomonadota, and to a lesser extent by Actinomycetota and Bacillota.

## 4. Discussion

The blooming of the microbiome studies during the last decades has brought to light the high taxonomic variability and functional stability in the bacterial communities in oceans, soils and holobionts [4,29,44,45,46,47,48]. Such observation is in line with our meta-analysis approach in which the bacterial variability disclosed within and between each one of the three analyzed feeding trials corroborated and extended the notion that the diet is a main regulator of the gut microbiota in fish [18,24,49,50,51]. Moreover, the inferred functional enrichment of bacteria taxons disclosed a similar KEGG (level 3) functional pattern regardless of trial in terms of the ratio and presence/absence of a given function (Figure 1). This is the usual pattern in humans [8,52,53], but also in plants, macroalgae and animal [10,54,55,56] bacterial–host associations, which suggests a similar evolutionary trend along all biological systems. This makes sense under a high variable host–microbiota scenario that has evolved to achieve a certain balance and stability at the same time, in order to guarantee the success in dealing with disturbing events [26,57,58]. In other words, very distant phylogenetically microorganisms could be able to express the same specific functions, meaning that a great taxonomical modification does not automatically mean a substantial remodeling of the bacterial metabolic capacity [9,11,54]. Certainly, when comparing marine-farmed fish strains of gilthead sea bream [16,18] and European sea bass [17] with a different growth potentiality, it appears that the gut microbial combination that provides a high microbiome taxonomic variability and functional redundancy is not the most advantageous and profitable situation in terms of fish performance. Therefore, the identification of the particular bacterial groups commanding the effects of a determined genetic or nutritional intervention arises as necessary for identifying the main factors regulating the microbial community. Our approach, based on a combination of core microbiota and BN calculation, represents one of the first steps towards this goal.

The definition of a healthy gut microbiota under a specific condition is now a recurrent topic in aquaculture, because the microbial community inhabiting within and surrounding farmed fish contributes directly to productivity in terms of growth, disease resistance and animal welfare [59,60,61]. However, our approach aimed to transcend the simple characterization of gut microbiota, passing through the identification of microbial associations that can account for the most relevant and active portion of the whole microbial community from a holobiont point of view. In the present study, the first step taken in this direction was to calculate the core microbiota shared from the three feeding trials, each one with different diet formulations but with some overlapping. Such approach rendered a highly conserved core microbiota across feeding trials, which was composed of 10 genera (*Vibrio*, *Bacillus*, *Pseudomonas*, *Sphingomonas*, *Acinetobacter*, *Corynebacterium*, *Clostridium*, *Staphylococcus*, *Enterococcus* and *Streptococcus*) after the application of restrictive filters (Figure 2). This list is in accordance with previous studies, which have indicated a core microbial population mostly composed by generalist taxa that might be able to use a wider range of resources and to occupy different niches and host compartments [25,62,63]. However, this consideration of core microbiota is limited to a taxonomic perspective and does not consider its real functional role within the community [26,27,29,64]. In this regard, one of the first definitions of functional core microbiota was introduced by Lemanceau and coworkers [65], who considered the microorganisms as mere vehicles of genes (replicators) that have the potential to exert essential functions with a main impact on the growth and health of the holobiont system. We aimed to go further with the BN approach, which highlighted the important functional role of the core microbiota acting as parents in most of the established hierarchical relationships. However, it remains elusive whether this is a constant trait on a temporal or spatial basis when considering different productive and/or biological sources of variation, as stated before by several authors [27,66]. Anyway, the study of the core microbiota represents an effective strategy for pinpointing a representative sub-group of the entire population. Moreover, within this subset, the identification of microbial associations and the effect of the diet variable was easier to compare and evaluate, reinforcing the importance of a small list of bacteria in the maintenance and regulation of the host–microbial cross talk, but also upon the control and stability of the entire bacterial community through the synthesis and processing of certain metabolites and signal molecules [27,67].

On a closer look, the separate identification of the core microbiota in each feeding trial shaped a relatively high similarity of NOPAP-PP and NOPAP-SCP fish groups, tearing apart the PAP group, which was mainly driven by the *Vibrio*, *Staphylococcus*, *Sphingomonas*, *Gordonia*, *Bradyrhizobium* and *Novosphingobium* genera (Figure 3 and Figure 4). These results are aligned to the gut microbial differences already reported when comparing fish fed with diets enriched in PAP and NOPAP ingredients [37,38,39]. Based on this evidence, a second approach recalculated two different BNs (one for NOPAP fish and one for PAP fish) including the effect of the variable diet in the resulting bacterial networks. Interestingly, both models exhibited the significant influence of the diet on the microbial community, connecting up to ~130 taxa representing more than the 50% of the total bacterial abundance (~70% in NOPAP; ~56% in PAP). Therefore, the introduction of the variable diet induced the re-calculation of the causal dependencies between bacteria, increasing the number of interconnected bacteria. Despite this, the core microbiota still played an important role at the hierarchical level in both PAP and NOPAP BNs, representing almost 50% of the total bacterial population (Figure 5). Moreover, the results showed that the core takes part in about half of the network relationships (47.5% in NOPAP; 51.3% in PAP BN).

As a general trend, the identification of the heavily connected variable is a crucial feature in network science due to its wider applicability and capacity to solve several associated problems, but also to visualize and quantify sub-groups of nodes, generally defined as modules or communities, which can have particular properties participating in dynamic processes [68,69]. In agreement with this, the Leiden clustering algorithm classified our annotated bacteria with strict stochastic relationships. Among them, the isolated nodes included in cluster 0 represented a low, but consistent (~10%), fraction of the intestinal microbiota not correlated with the variable diet. This cluster contained bacteria belonging to the *Photobacterium*, *Propionibacterium* and *Actinomyces* genera in the NOPAP BN; and *Psychrobacter*, *Actinobacillus* and *Bacteroides* in the PAP BN. The presence of this bacteria is a common feature in a wide range of species, including Atlantic salmon (*Salmo salar*), Atlantic cod (*Gadus morhua*), gilthead sea bream and fine flounder (*Paralichthys adspersus*), though our results might suggest that their presence is mostly independent of the nutritional background [37,38,70,71,72,73,74,75]. The role of these nodes in the gut microbial network is still under evaluation and clarification, but they remained separate from the principal hierarchical structure. Conversely, the rest of the clusters that encompassed bacteria with the variable diet become especially relevant for evaluating the nutritionally regulated microbiota, which emphasized the ability of these bacterial associations to potentially express or take part in specific metabolic pathways not necessarily distributed equally within a larger bacterial population. Following this pattern, some functions were widely distributed among different bacteria clusters (Table 1). Interestingly, overlapping KEGG pathways were also found between PAP and NOPAP BNs, which referred among others to flavone and flavonoid biosynthesis, bile secretion, the relaxin signaling pathway, retrograde endocannabinoid signaling and staurosporine biosynthesis (Figure 6a). Whitin this list, those functions related to the metabolism of bile salts and flavonoids are part of the bacteria–host associations. Thus, numerous microbial taxa, including members of lactic acid bacteria, and the genera *Clostridium* and *Bacteroides* [76], have been shown to be able to hydrolyze and oxidize primary bile acids through the action of stereospecific hydroxysteroid dehydrogenases [77]. In addition, strictly anaerobic bacteria, such as the genus *Clostridium* XIVa and XI, are capable of further biotransformation of bile acids, rendering the formation of secondary bile acids derivates. These molecules are important for the intestinal homeostasis, as they can be used by the host for nutritional purposes, but also because they act as modulators of the bacterial populations, both promoting the bacteria capable of metabolizing these substrates and inhibiting the intestine colonization by other microbial competitors [78]. Likewise, gut microbiota can play an important role in the metabolism of flavonoids, contributing to activating these compounds and making them more bioavailable to the host. Certainly, studies in humans have demonstrated that the resulting end products help to improve intestinal health and mitigate metabolic disease [79,80]. Similar positive results have been documented in fish, where the addition of flavonoids in the diet promoted growth and flesh quality, reducing the risk of oxidative stress [81,82].

According to all the above findings [16,83,84,85], a full comprehension of the gut microbiota function in fish is still far away [16,83,84,85]. This gap in the knowledge is intrinsic to the microbiota variability, which complicates the measurement of the real contribution of each bacterial population to the host’s physiology. Despite this, some promising results were described herein for some of the metabolic pathways, which resulted in positive correlation with abundant taxa like *Vibrio*, *Photobacterium* and *Propionibacterium* [37,38,39]. Similar relevant host–bacteria associations have been observed by Piazzon et al. (2020) and Naya-Català et al. (2022), who described a better physiological adaptation and a more plastic microbiota in genetically selected gilthead sea bream families for enhanced growth [16,18]. The microbial community can, in fact, be an effective actor in many biochemical reactions that can have local effects or reach distant organs, such as the nervous system, modulating in turn the behavioral patterns and contributing to the success of the holobiont fitness [86,87,88,89]. However, in line with a largely documented functional redundancy [7,11,54], our results highlighted that almost all the microorganisms associated to a given function were clearly different in the PAP and NOPAPA BNs. This main outcome confirmed the metabolic plasticity of the bacteria, but also unveiled the specific effect of the PAP and NOPAP diet formulations in the modulation and reorganization of the whole microbiota population. However, detailed indications on the association between bacteria and diet are difficult to find in the literature as they are scarce and fragmentary. Several authors have in fact described changes in some microbiota taxa focusing more on changes in the proximal composition of feed, or hardly reaching low taxonomic levels, such as genera or species [90,91]. In this context, the two different lists of bacteria in Figure 7 represent a step forward in the discovery of a strong link that joins the presence/abundance of certain microorganisms with a given feed ingredient. These groups in fact contain bacteria associated with NOPAP (e.g., *Vibrio*, *Marinomonas*, *Propionibacterium*, *Brevundimonas*, *Staphylococcus*), and PAP feeds (e.g., *Tetrasphaera*, *Paracoccus*, *Bacillus*, *Clostridium* XIVa, *Streptococcus*), in addition to some bacteria taxa (*Acinetobacter*, *Bacillus*, *Arthrobacter* and *Enterobacter*) previously identified as core microbiota. Ultimately, their strict stochastic dependency with the diet makes these genera the first candidates to be defined as reliable microbiota biomarkers, combining taxonomy classification, community hierarchy and functional contribution. From this perspective, these results emphasize the need to increase the number of pivotal prokaryotic indicators associated with precise farming conditions. To achieve this goal, corroborate the present results and obtain more robust models and predictions, it will be necessary to feed the system with more data. Further development of this work will rely on specific experimental setups, designed to emphasize the effects of environmental variables important for aquaculture, such as water temperature, salinity and oxygen level, and considering longitudinal studies to reveal the temporal evolutions of microbiota. Finally, the ultimate step in this research will consider the integration of metagenomic, transcriptomic and metabolomic data, using SAMBA as a multi-omics platform, to discover in detail the intimate connections between microbiota, host, diet and environment.

## 5. Conclusions

The results of this study, from a computational and a meta-analysis perspective, highlighted the high taxonomical variability and functional redundancy of the intestinal microbiota composition of gilthead sea bream, bringing to light these intrinsic characteristics also in the aquaculture sector, in which these features are still underestimated. Although very useful in a natural environment, the highest microbial biodiversity and functional plasticity may not be the most profitable scenario in a producing farming condition. For this reason, the identification of the most influential multi-factorial markers within the microbiota population is crucial in understanding the interconnection between bacteria and host and to maximize the benefits. To achieve this goal, the approach used in this work was developed around the definition of a robust core microbiota and the construction of Bayesian networks. The achieved results confirmed the crucial importance of the core microbiota as an effective synthesis unit of the whole microbiota populations, also introducing an innovative protocol to determine the structural hierarchy and the functional profiles of the different clusters of bacteria in response to a distinct nutritional background. Several and repetitive applications of this protocol will ultimately reinforce the comprehension of the cross-talk within the holobiont system, integrating the contribution of the healthy core microbiota and the novel microbiota biomarkers, associated with precise farming and environmental conditions, but defined using a new paradigm which combine taxonomy, community organization and functional features. This sequence of processes makes the present perspective a useful improvement for increasing the knowledge about fish intestinal microbiota. Such new information, together with their future experimental and empirical confirmations, will fill the existing gaps in aquaculture, allowing further development of the sector through the modulation and investigation of those small differences in the fish microbiome that functional redundancy and taxonomic variability can mask.

## Figures and Tables

**Table 1:** List of significant (*p*-adjusted < 0.05) functional enrichments obtained for both NOPAP and PAP models and divided by the clusters that express them.

NOPAP						
Clusters	Level 2	Description	Ratio	Bg Ratio	*p* Value	*p*. Adjust
**2**	Biosynthesis of other secondary metabolites	**Flavone and flavonol biosynthesis**	5/19	182/3364	0.003	0.033
	Biosynthesis of other secondary metabolites	Biosynthesis of various alkaloids	3/19	58/3364	0.004	0.037
	Folding, sorting and degradation	**Proteasome**	8/19	440/3364	0.002	0.026
	Immune system	**RIG-I-like receptor signaling pathway**	10/19	420/3364	<0.001	0.001
**5**	Biosynthesis of other secondary metabolites	**Staurosporine biosynthesis**	5/11	239/3364	0.001	0.014
	Biosynthesis of other secondary metabolites	Isoflavonoid biosynthesis	3/11	95/3364	0.003	0.022
	Digestive system	**Bile secretion**	5/11	183/3364	<0.001	0.011
	Endocrine system	**Relaxin signaling pathway**	2/11	12/3364	0.001	0.014
	Endocrine system	**Parathyroid hormone synthesis, secretion and action**	4/11	146/3364	0.001	0.014
	Endocrine system	**Melanogenesis**	3/11	84/3364	0.002	0.021
	Immune system	**Platelet activation**	2/11	22/3364	0.002	0.021
	Infectious disease: bacterial	**Yersinia infection**	3/11	107/3364	0.004	0.026
	Metabolism of terpenoids and polyketides	Biosynthesis of type II polyketide products	3/11	136/3364	0.008	0.044
	Nervous system	**Retrograde endocannabinoid signaling**	3/11	97/3364	0.003	0.022
	Signaling molecules and interaction	ECM-receptor interaction	2/11	36/3364	0.006	0.033
**7**	Immune system	**RIG-I-like receptor signaling pathway**	11/33	420/3364	0.001	0.037
	Biosynthesis of other secondary metabolites	**Flavone and flavonol biosynthesis**	7/33	182/3364	0.002	0.037
**8**	Biosynthesis of other secondary metabolites	Indole alkaloid biosynthesis	5/11	212/3364	<0.001	0.013
**PAP**						
**1**	Folding, sorting and degradation	**Proteasome**	9/17	440/3364	<0.001	0.004
	Digestive system	**Bile secretion**	6/17	183/3364	<0.001	0.004
	Biosynthesis of other secondary metabolites	Biosynthesis of various other secondary metabolites	7/17	331/3364	0.001	0.009
**3**	Endocrine system	**Parathyroid hormone synthesis, secretion and action**	4/10	146/3364	0.001	0.034
**4**	Cell growth and death	Cellular senescence	2/10	20/3364	0.001	0.009
	Circulatory system	Vascular smooth muscle contraction	2/10	20/3364	0.001	0.009
	Circulatory system	Adrenergic signaling in cardiomyocytes	2/10	38/3364	0.005	0.015
	Digestive system	Gastric acid secretion	2/10	33/3364	0.004	0.013
	Digestive system	**Bile secretion**	3/10	183/3364	0.014	0.035
	Endocrine system	Regulation of lipolysis in adipocytes	3/10	25/3364	<0.001	0.002
	Endocrine system	**Melanogenesis**	4/10	84/3364	<0.001	0.002
	Endocrine system	**Relaxin signaling pathway**	2/10	12/3364	0.001	0.006
	Endocrine system	Oxytocin signaling pathway	3/10	64/3364	0.001	0.008
	Endocrine system	GnRH signaling pathway	3/10	72/3364	0.001	0.009
	Endocrine system	Renin secretion	4/10	209/3364	0.002	0.009
	Endocrine system	Aldosterone synthesis and secretion	2/10	32/3364	0.004	0.013
	Environmental adaptation	Circadian entrainment	2/10	26/3364	0.002	0.009
	Immune system	**Platelet activation**	2/10	22/3364	0.002	0.009
	Immune system	**RIG-I-like receptor signaling pathway**	5/10	420/3364	0.004	0.014
	Immune system	C-type lectin receptor signaling pathway	2/10	38/3364	0.005	0.015
	Nervous system	Cholinergic synapse	2/10	22/3364	0.002	0.009
	Nervous system	Long-term potentiation	2/10	25/3364	0.002	0.009
	Nervous system	**Retrograde endocannabinoid signaling**	3/10	97/3364	0.002	0.009
	Sensory system	Inflammatory mediator regulation of TRP channels	2/10	22/3364	0.002	0.009
	Signal transduction	Ras signaling pathway	3/10	68/3364	0.001	0.008
	Signal transduction	NF-kappa B signaling pathway	2/10	25/3364	0.002	0.009
	Signal transduction	TNF signaling pathway	2/10	25/3364	0.002	0.009
	Signal transduction	VEGF signaling pathway	2/10	26/3364	0.002	0.009
	Signal transduction	Apelin signaling pathway	2/10	38/3364	0.005	0.015
	Signal transduction	Calcium signaling pathway	3/10	151/3364	0.008	0.022
	Signal transduction	Sphingolipid signaling pathway	3/10	170/3364	0.012	0.03
**5**	Xenobiotics biodegradation and metabolism	Bisphenol degradation	2/3	68/3364	0.001	0.043
	Infectious disease: bacterial	**Yersinia infection**	2/3	107/3364	0.003	0.043
**6**	Lipid metabolism	Steroid biosynthesis	4/6	259/3364	<0.001	0.012
**7**	Biosynthesis of other secondary metabolites	**Flavone and flavonol biosynthesis**	3/4	182/3364	0.001	0.011
**8**	Biosynthesis of other secondary metabolites	**Staurosporine biosynthesis**	5/13	239/3364	0.001	0.043
**9**	Transcription	Spliceosome	1/3	18/3364	0.016	0.048

## Data Availability

All the microbiota datasets used in this study can be found online associated with their respective publications [37,38,39].

## Supplementary Materials

The following supporting information can be downloaded at: https://www.mdpi.com/article/10.3390/microorganisms13010198/s1, Figure S1: graphical representation of the validation of the PLS-DA model by random permutations. Representation of the contribution of each component to variance explained (R2Y) and predicted (Q2), driving the separation of the three experiments (NOPAP-PP, PAP, NOPAP-SCP); Table S1: ingredients and chemical composition of experimental diets used in the three different feeding trials used (NOPAP-PP, PAP, NOPAP-SCP), as reported in the published works [37,38,39]; Table S2: complete list of taxa represented in Figure 1a reporting their relative abundance in each experimental groups within each experiment (NOPAP-PP, PAP, NOPAP-SCP) and the *p*-value, obtained by Kruskal–Wallis test between groups; Table S3: results of DESeq analysis carried out on the inferred metagenomic functional enrichment analysis of the three experiments (NOPAP-PP, PAP, NOPAP-SCP), using the whole microbiota populations. The results are reported for each comparison within each experiment and divided by both level 2 and level 3 of KEGG pathway description assignations; Table S4: complete list of nodes representing the core microbiota within the merged BN reported in Figure 3, classified by the categories (A; B; C) showed in Figure 2c, and reporting their relative abundances in each experiment (NOPAP-PP, PAP, NOPAP-SCP); Table S5: complete list of nodes and edges of both BNs (NOPAP; PAP) illustrated in Figure 5a,b. The nodes report the core microbiota category, the number of clusters of which it is part, the centrality degree (C. Degree), the value of parent (Par %), and its total average abundance. The edges report the parent taxa and the child taxa, indicating for both the cluster of which each node is part, the BIC, MI value and the score which together define the strength of each causal dependency of both models.
