# Supplementary material for: Beyond Microbial Variability: Disclosing the Functional Redundancy of the Core Gut Microbiota of Farmed Gilthead Sea Bream from a Bayesian Network Perspective
Source: Microorganisms. 2025 Jan 17;13(1):198. doi: 10.3390/microorganisms13010198 (PMC11767429; doi:10.3390/microorganisms13010198)
Supplement: Supplementary file 1 [file microorganisms-13-00198-s001.zip › Supplementary Files revised/Table S1.pdf]

**NOPAP-PP**			
**Ingredients**	CTRL (%)	L-FM/FO (%)	EWH (%)
Fishmeal super prime	30	10	10
Fish protein hydrolysate	5	2.5	
Soy protein concentrate	12.5	12.5	10
Pea protein concentrate		4.5	2.5
Wheat gluten	5	12.5	10
Corn gluten meal	5	10	10
Soybean meal	5	10	10
Rapeseed meal	5	5	
Sunflower meal	5	10	10
Wheat meal	9.07	3.99	5.49
Whole peas	4	2	2
Vitamin and mineral	1	1	1
Vitamin C35	0.03	0.03	0.03
Betaine HCl	0.2	0.2	0.2
Antioxidant powder	0.2	0.2	0.2
Sodium propionate	0.1	0.1	0.1
Monocalcium phosphate	0.6	2.5	2.5
L-Tryptophan		0.03	0.03
DL-Methionine		0.25	0.25
Fish oil	5	3	3
Soybean oil	5.3	8	8.6
Linseed oil	2	1.7	1.6
EGG hydrolysate			7.5
**Chemical composition (proximate analyses)**			
Dry matter, % feed	92.9	93.2	92.8
Crude protein, % feed	47.9	47.9	47.9
Crude fat, % feed	16.1	16.1	16.1
EPA C DHA, feed	2.4	1.2	1.2
Ash, % feed	8.7	8	7.6

**PAP**			
**Ingredients**	CTRL (%)	50LSAqua (%)	100LSAqua (%)
Fish meal, herring, 70% crude protein	15	7.45	
Gluvital1	10.4	11	11
Guar korma	12	12	11.9
Wheat	11	11.9	11
Corn gluten 60	12.5	10	10
DHA oil	7.5	7.86	8.2
Rapeseed oil	6.3	6.22	6
Rapeseed	7	7	7
Soycomill R2	6	5	5
Bosoy – GMO3	6	3.8	3
Bicaphosph 18 P/25 Ca2	2	2.6	3.35
PMX Fish5	2	2	2
Alphasoy 530 GMO6	2	1.5	1.1
L-lysine HCl	0.28	0.088	0.137
Limestone Ca		0.067	0.07
DL-methionine	0.007	0.105	0.193
Salt, 99% NaCl	0.013	0.11	0.25
LSAqua SusPro		11.3	19.8
**Chemical Composition**			
Crude protein, % feed	43.09	43.312	43.768
Crude fat, % feed	18.434	18.203	18.021
EPA + DHA, % feed	2.214	2.216	2.214

**NOPAP-SCP**		
**Ingredients**	CTRL (%)	NoPAP SANA (%)

Fish meal Super Prime	10	
Fish meal by-products 7	7	
Fish protein hydrolysate 3	3	
Fish protein hydrolysate aquaculture		3
Poultry meal 10	10	
Insect meal (Black soldier		10
Fermentation biomass (Corynebacterium glutamicum)		5
Fermentation biomass (Methylococcus capsulatus)		10
Soy protein concentrate 6	6	4.5
Pea protein concentrate		6.1
Wheat gluten 4 3	4	3
Corn gluten meal 10	10	7.5
Soybean meal 48 12.5	12.5	
Rapeseed meal	7	
Sunflower meal 40	5	20
Wheat meal	10.61	
Whole peas		6.12
Pea starch (raw)		3.6
Vitamin & Min Premix	1	
Vitamin & Min Premix		1
GAIN Macroalgae SHP3		2.5
GAIN Macroalgae SHP Se-rich4		0.1
GAIN Microalgae WUR Se-rich5		0.2
Vitamin E50	0.03	0.03
Betaine HCl	0.1	0.1
Antioxidant6	0.2	0.2
Sodium propionate	0.08	0.08
Monoammonium phosphate	0.55	2.6
L-Lysine HCl 99%		0.3
L-Threonine		0.05
L-Tryptophan	0.06	0.1
DL-Methionine	0.05	0.3
Yttrium oxide	0.02	0.02
Fish oil	4.9	2.5
Salmon oil by product		9.6
Algae oil (Veramaris) 1		1
Rapeseed oil	7.9	
SANACORE7		0.5
**Composition analysis (% dry matter)**		
Protein	50.5	49.6
Lipid	17	17.6
Ash	8.3	7.2
Total P	1.6	1.8
EPA + DHA	1.9	2
Energy (MJ/kg dry matter)	22.3	22.6
