# Supplementary figures and images for: Beyond Microbial Variability: Disclosing the Functional Redundancy of the Core Gut Microbiota of Farmed Gilthead Sea Bream from a Bayesian Network Perspective
Source: Microorganisms. 2025 Jan 17;13(1):198. doi: 10.3390/microorganisms13010198 (PMC11767429; doi:10.3390/microorganisms13010198)

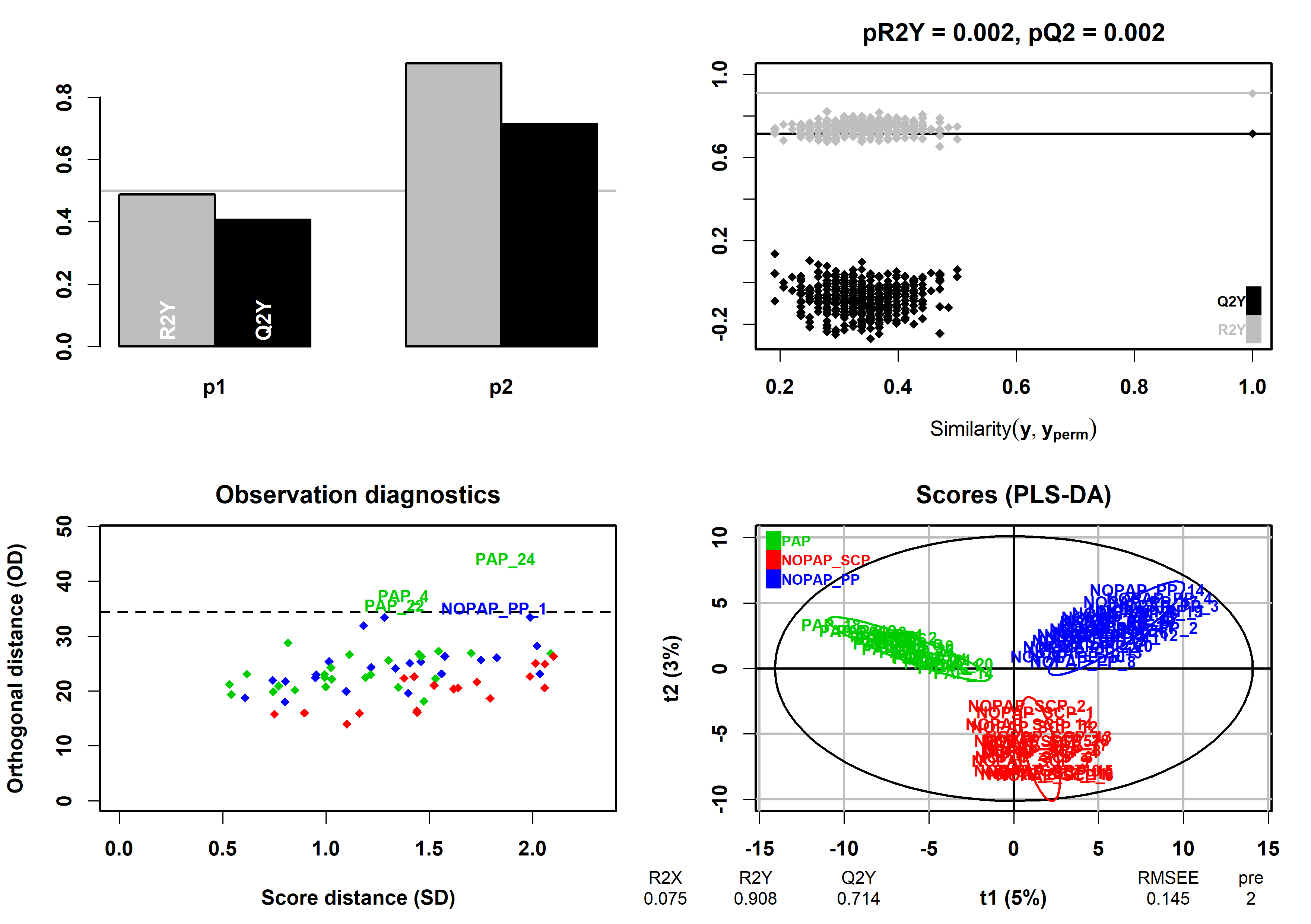

Supplement: Supplementary file 1 [file microorganisms-13-00198-s001.zip › Supplementary Files revised/Figure S1.tiff]
